# Challenges and opportunities in hydroxyurea access and adherence for sickle cell disease patients at Komfo Anokye Teaching Hospital: Insights from healthcare providers

**DOI:** 10.1371/journal.pone.0347848

**Published:** 2026-05-29

**Authors:** Josephine Mensah, Afia Frimpomaa Asare Marfo, Mercy Naa Aduele Opare-Addo, Paul Obeng, Amos Amoako-Adusei, Karl Osei Afoduo, Bright Ansah Adjei, Pauline Boachie Ansah, Kwame Ohene Buabeng

**Affiliations:** 1 Pharmacy Directorate, University of Ghana Medical Centre Ltd, Legon, Accra, Ghana; 2 Department of Pharmacy Practice, FPPS, Kwame Nkrumah University of Science and Technology, Kumasi, Ghana; 3 Department of Pharmacy, Komfo Anokye Teaching Hospital, Kumasi, Ghana; 4 Kumasi Centre for Collaborative Research in Tropical Medicine, Kumasi, Ghana; 5 Medical and Scientific Research Centre, University of Ghana Medical Centre Ltd, Legon, Accra, Ghana; Versiti Blood Research Institute, UNITED STATES OF AMERICA

## Abstract

Hydroxyurea is a vital medication for managing Sickle cell disease, yet access and adherence remain significant challenges in low-resource settings. This qualitative study explored challenges and opportunities in hydroxyurea access and adherence among Sickle cell disease patients at the Komfo Anokye Teaching Hospital in Ghana. Alongside, the utility of the Ahodwo CommCare mobile App in enhancing Sickle cell disease management was explored. Semi-structured interviews were conducted with six healthcare providers, including doctors, nurses, and pharmacists from May 12 to August 15, 2022. This study found that the key barriers to hydroxyurea access and adherence include financial constraints, medication stock-outs, administrative inefficiencies, bureaucratic delays, patient-related factors such as forgetfulness and concerns about side effects, and practical challenges in administering the medication especially to children. The healthcare workers indicated that reconstituting the medication into suspension added both cost and time constraints to children who cannot take capsules hence affecting access to hydroxyurea. Facilitators to access and adherence identified included patient education, streamlined procurement processes, and government support for medication subsidies. This study found that patient education sessions should emphasize the benefits of hydroxyurea treatment and the importance of adherence to prescribed regimens. The study also revealed limited awareness and utilization of the Ahodwo CommCare app among healthcare personnel, highlighting the need for targeted training and integration into clinical workflows. These findings underscore the importance of multifaceted interventions involving healthcare workers, hospital management, and government stakeholders to improve hydroxyurea access and adherence, ultimately enhancing Sickle cell disease patient outcomes.

## Introduction

Sickle cell disease (SCD) is a significant public health challenge in sub-Saharan Africa, with Ghana bearing a high burden of the disease [1–4]. Hydroxyurea (HU) has proven to be an effective therapy for reducing complications and improving the quality of life for SCD patients [5–10]. HU is a myelosuppressive agent which provides therapeutic benefits in the management of SCD across all ages and is readily absorbed upon oral administration [11]. During the first four to six weeks of administering HU, the mean cell volume (MCV) increases, before the cells bearing Fetal Hemoglobin (HbF) expand, a situation that is associated with decreases in vaso-occlusive crisis (VOC) events [12]. However, access and adherence to HU remain suboptimal due to systemic, facility-related, and patient-level barriers [8,13–17]. Several factors have been identified as the cause of high SCD related deaths and reduced quality of life in these settings. Some of the factors include unavailability of established programs for neonatal SCD screening, poorly managed neonatal screening programs for SCD, inadequate information sharing and education of stakeholders about successful evidence-based treatment options, poor access to required medications and essential clinical support services, and limited healthcare facilities [1].

Adherence of patients to medications is a dynamic process which is influenced by a myriad of factors from three different perspectives: patient, healthcare providers (HCPs), and healthcare system [18]. Understanding these barriers is imperative to developing targeted interventions that can improve medication access and adherence, particularly in resource-limited settings [19].

Several interventions have been employed to enhance medication adherence in patients with chronic illnesses [20,21]. Electronic devices have been shown to be useful in facilitating provision of interventions targeted towards improvement of medication adherence and disease self-management in children and adolescents who have chronic diseases such as SCD [22,23].

The use of interventions based on technology is a common occurrence in health care in recent times. The use of mHealth interventions has been linked to improved patient engagement and self-management of health, thus making them an applicable option to be employed in the quest to achieve improved patient outcomes [24,25]. Several studies conducted have shown that SCD patients and caregivers preferred to use mHealth technologies for management of issues pertaining to SCD and other patient related factors [26,27].

Educating healthcare workers (HCWs) on the benefits of HU, guidelines for its administration, standardized prescription practices, and patient counselling can be a valuable strategy to enhance HU acceptance.

This study was conducted at the SCD clinic of Komfo Anokye Teaching Hospital (KATH), a major referral centre in Ghana. The primary aim was to identify patient, system, and facility-related factors limiting HU access and adherence, as well as to explore the potential of the Ahodwo CommCare mobile app in enhancing SCD management. By engaging HCPs through qualitative interviews, the study sought to capture their perspectives on barriers to HU adherence, strategies for improvement, and the role of digital health tools in patient care. The findings from this study contribute to the growing body of literature on SCD management in low-resource settings and provide actionable insights for stakeholders aiming to improve HU access and adherence. By addressing systemic barriers and leveraging digital health solutions, it is possible to enhance patient outcomes and reduce the burden of SCD in affected communities.

## Method

### Study design

This study involved qualitative interviews conducted with healthcare personnel at the SCD clinic, KATH. The primary aim was to identify the patient, system, and facility related factors limiting the access and adherence to HU by SCD patients and explore the usefulness of the current mobile App available for management of SCD patients at the clinic.

### Study Setting

This study was conducted at the SCD clinic of KATH located in Kumasi, the capital of the Ashanti Region. The hospital serves a population of approximately 3,903,000. As the second-largest hospital in Ghana, it receives referrals from the northern half of the country due to its strategic geographical location. The hospital’s accessibility is enhanced by the region’s extensive road network and Kumasi’s commercial nature. With a capacity of 1,200 beds, the hospital operates as a Teaching Hospital, affiliated with the Kwame Nkrumah University of Science and Technology’s School of Medical Sciences. The hospital’s organizational structure comprises 15 directorates, including 13 clinical and 2 non-clinical directorates, supported by various clinical and non-clinical units. The KATH SCD clinic is one of the most established specialized centres for SCD care in Ghana. The KATH Child Health Directorate runs the specialist outpatient clinic 5 days a week and a 24hour inpatient service. A multidisciplinary team of paediatric haematology experts including medical doctors, pharmacists and nurses provides specialized care for the patients [28]. As a key referral centre, the clinic offers comprehensive services, including newborn screening, chemoprophylaxis, vaccinations, and disease-modifying therapies such as HU medication to over 4,000 SCD patients from across the country annually, particularly the central and northern regions [17].

### Study participants and sampling

Participants for this study included all HCWs at the SCD clinic. During the data collection period, a total of seven HCWs attended to patients at the SCD clinic, and a convenient sampling method was used to select participants which included two medical doctors, three nurses, and one pharmacist. There were no specific exclusion criteria beyond lack of consent.

### Data collection

To guide the in-depth interview (IDI) of HCWs at the SCD clinic, a semi-structured questionnaire was developed. This questionnaire incorporated both open-ended and closed-ended questions, designed to gather information on several key aspects: the availability and use of HU in the clinic, the perceived barriers to its utilization and the personal and institutional factors that influence its prescription and use. The semi-structured format was particularly advantageous as it allowed for flexibility, enabling interviewers to delve deeper into specific issues that participants raised. This approach not only facilitated the collection of rich qualitative data but also ensured that the interviews were dynamic and responsive to the unique insights of each participant. Data collection spanned from May 12 to August 15, 2022. During this period, qualitative interviews were conducted with selected HCPs, including medical doctors, nurses, and a pharmacist. Each interview was scheduled at a time that was convenient for the participants, ensuring minimal disruption to their professional duties. This helped to create a relaxed environment where participants felt comfortable and were more likely to share responses without external influences. This setting was necessary in encouraging open communication, as participants could speak freely without concerns about being overheard or interrupted. All participants provided written informed consent. The HCWs were given consent forms, which they completed and signed. To ensure the accuracy of the data collected, all interviews were audio-recorded with the participants’ informed consent. This practice not only captured the exact words of the respondents but also allowed for the preservation of differences in tone and emphasis, which are often important in qualitative research.

### Data processing and analysis

The combination of semi-structured interviews, audio recording, and thematic analysis provided a robust method for understanding the complex factors influencing the availability and use of HU in the clinic. This approach ensured that the voices of various healthcare professionals were captured and that the findings were grounded in their lived experiences and professional insights. All audio-recorded interviews were transcribed verbatim to create textual data for analysis. Transcriptions were reviewed for accuracy by cross-checking with the audio recordings. The transcribed data were systematically coded using both inductive and deductive approaches. Codes were generated to identify key themes and sub-themes related to the availability and barriers to the use of HU. Coded data were organized into themes that reflected recurring patterns, significant insights, and notable issues raised by the participants. Themes were developed around the main research questions and objectives. An analytic framework was utilized to compare and contrast the different perspectives of doctors, nurses, and pharmacists, highlighting commonalities and differences in their experiences and perceptions. To ensure the reliability and validity of the findings, member checking was performed. Participants were provided with a summary of the findings to confirm the accuracy of the data interpretation.

### Ethical considerations

Ethical clearance was obtained from the KATH Institutional Review Board (KATH IRB AP/128/21) prior to the commencement of the study. Consent of participants were sought, and only those who agreed were included in the study. Participant information leaflets were developed explaining to the prospective participants the research objectives, risks and benefits of being enrolled in the study. Participants were also given the opportunity to ask questions to ensure they understood the study objectives very well. Participants were informed that enrolling into the study was voluntary, and that they could withdraw from the study at any given time. All participants provided written informed consent. The HCWs were given consent forms, which they completed and signed. Consent was also sought for from the Head of the Child Health Directorate, KATH before the study was commenced.

## Results

This study involved six HCWs who were directly engaged in the management and care of patients with SCD during the study period. The demographic profile of the respondents revealed a sex distribution of two [2] males and four [4] females. Participants represented a range of healthcare professions, including medical doctors, nurses and pharmacists. The age distribution of the respondents showed a mean age of 33.75 + /- 6.16 years (range: 28–44years). Notably, two of the respondents opted not to disclose their age. In terms of religious affiliation, all six participants identified as Christians. The respondents’ professional experience was characterized by a mean of 8.17 + /- 3.85 years of practice in their respective healthcare professions (range: 3–15years). Specifically, their experience in the SCD clinic setting averaged 5.83 + /- 4.33 years (range: 1–15years). This diversity in experience and profession provided a comprehensive perspective on the care and management of patients with SCD.

### Barriers to hydroxyurea adherence

Respondents of the study revealed several key challenges that impact the consistent use of the medication. These included financial constraints, medication availability issues, practical challenges in administration, forgetfulness, and concerns about side effects. One of the most prominent barriers identified by healthcare providers and patients alike was the financial burden associated with acquiring HU.

*“The main barrier has to do with finances, because previously the medication wasn’t insured, so patients had to buy out of pocket. And you know, in our parts of the world, especially in sub-Sahara Africa... most patients usually find it difficult to raise money for this expensive drug. The next barrier will be access in terms of where patients find the medication because they do not find it all the time within the hospital and so they must go outside for it” (Respondent 1, Male HCW)*

*“Money is the main barrier to the administration of the drug... Sometimes when patients are not able to come for appointments, they stop taking the drug, especially when the medicine gets finished along the way, they stop taking the medicine until they come back for the doctor to prescribe...” (Respondent 6, Female HCW)*

*“Another barrier to adherence is the frequent lab investigations that come with HU therapy. For some parents, when it comes to sticking needles into their children, they are quite wary. And then based on that, they wouldn’t want to be bringing their children quite frequently for lab investigations. So that’s also a barrier to adherence. And of course, HU, even though it’s supposed to be on the National Health insurance scheme, we don’t usually have continuous or regular supply. So, patients who are not able to afford it are not able to get it when they run out of supply… looking at where Ghana is located, low middle income country, financial challenges, is one of the main barriers…” (Respondent 6, Female HCW).*

Additionally, another male HCW also expressed his sentiments as follows:

*“For now, the only place you can access HU is the specialist OPD pharmacy... the scope should be widened. Other directorates or units should also be given the opportunity to serve these medications so that patients won’t queue for a very long time before they can access HU for free” (Respondent 5, Male HCW)*

Furthermore, difficulties associated with administering HU presents additional challenges, especially for children who cannot take capsules. Additionally, forgetfulness among patients, lack of clear instructions, and perceived side effects contribute to non-adherence, and these were also cited as challenges.

*‘’For children, we need to reconstitute the medication into suspension and this adds both cost and time constraints, as patients must queue at the pharmacy for this process” (Respondent 3, Female HCW)*

*“Sometimes when patients are not able to come for appointments, they stop taking the drug, especially when the medicine gets finished along the way, until they come back for the doctor to prescribe...” (Respondent 2, Female HCW)*

*“…Some felt that they were having some kind of side effects of the medicine, and so they stopped taking it. Others were simply confused because they didn’t get the right instructions, so they didn’t come back for a refill when their first month or their initial supply was done” (Respondent 1, Male HCW).*

HCPs further highlighted issues of inadequate medication supply, particularly concerning patients covered by insurance. Despite the medication being covered under the National Health Insurance Scheme (NHIS), patients often encountered situations where HU was unavailable at pharmacies within the hospital, necessitating purchase from external pharmacies.

*“…Inadequate supplies, especially for patients with insurance, they get to the pharmacy and the drugs are not available. Patients are told that the drug is covered under the health insurance but when the patients come and the medication is prescribed for them, they are told that the drugs are not available, hence they should buy the drugs outside the hospital. The patients are then directed to pharmacies outside the hospital. So, you can imagine the stress. The patients may not even go and buy the drug” (Respondent 3, Female HCW).*

*“I don’t know how the government is funding HU drugs, but I believe the funding is not coming in as is supposed to because sometimes when the medication is made available at the facility, in less than three weeks, it gets finished. That means whatever amount they bought was not enough because in a week we attend to more than 200 patients. And if in just three weeks everything is finished then it means that there is not enough supply” (Respondent4, Female HCW).*

### Improving access and compliance to hydroxyurea

Improving access and compliance to HU among patients with SCD requires multifaceted interventions involving HCWs, hospital management, and government stakeholders. The responses from HCPs during the interview sessions offered valuable inputs into potential strategies for enhancing access and adherence to hydroxyurea treatment.

### Healthcare workers’ role

The HCW suggested that patient education sessions should emphasize the benefits of HU treatment and the importance of adherence to prescribed regimens. Furthermore, other HCW roles cited were advocacy efforts directed towards hospital management to ensure consistent availability of HU. Additionally, HCWs, particularly pharmacists and administrators have a role in addressing practical barriers to access such as medication stock-outs and long waiting times for patients to receive HU prescriptions

*“I’m sure we can only advocate to both patients and communities, and tell them about the benefits of HU. We can also advocate to the hospital management that beyond the beyond patient-related clinical benefits, reduction in frequent hospital visits also means that there is lower cost of care for the hospital. So, we need to do more advocacy” (Respondent 1, Male HCW).*

*“I think we have to let the patient know the importance of taking the drugs. Well, if the patient knows the importance of taking the HU, I think he or she will always adhere to the drug. I think that’s what the health worker can do because you can’t buy the drugs for the patients. We can’t do anything about it unless you tell the patient the importance of giving them the drugs” (Respondent 3, Female HCW).*

*“In terms of access, I mean, it’s basically IEC, you give them the information, you educate them and of course you communicate to them. When you communicate with them, that’s when you know if they have some myths or misconceptions when it comes to HU adherence and then you actually try to tackle those misconceptions. As a HCP, you should also make the environment where the patients are attended to as friendly as possible so that they will be more comfortable to come. and feel at ease to share their thoughts and communicate with us. This will enable us to help them and educate them so that they will adhere to their medications” (Respondent 6, Female HCW).*

### Hospital management’s responsibilities

To improve access, hospital management should prioritize timely procurement and distribution of HU to hospital pharmacies. Bureaucratic barriers that delay the availability of medication to patients must be identified and addressed promptly. Moreover, efforts should be made to negotiate favourable pricing agreements with suppliers to make HU more affordable for patients.

*“The hospital management, once the medicine is received on the premise should cut down on some of the bureaucracies and make the medication available to the pharmacies. This will ensure that when the patient goes to the pharmacies, they will have access to them. Sometimes the drug may be available, but it will take about a week before they are distributed to the pharmacies. Thus, if a patient comes within that week, they won’t have access to the medication. And once the patient leaves the hospital, they are gone until their next review” (Respondent 4, Female HCW).*

*“First of all, I know HU is FDA approved in Ghana. And at first, we used the Novartis brand. Now there are other brands in the system. So, you know, in Ghana, most things are kept based on money and sometimes we may even be putting money first instead of quality. So, I think we should be able to outsource HU from world-renowned or acceptable brands. Because some brands may be toxic to patients’ health. Those things can be looked at. Also, we have to make sure that patients have access to HU all year round. So, it should be stocked, and then we should anticipate when it will run out so that we don’t run out of stock. As a health facility, those are some of the things that we can also do to ensure that patients have access to medication” (Respondent 6, Female HCW).*

This study revealed that, expanding the scope of HU distribution beyond the specialist outpatient department (OPD) can enhance accessibility for patients, reducing waiting times and travel burdens associated with accessing medication. Hospital management should consider decentralizing HU distribution to other units or clinics within the hospital to improve convenience for patients.

*“…Ok, so for now, the only place you can access it is the specialist OPD. OK. So, I think to improve access the scope should be widened so that other directorates or units are also given the opportunity to serve these medications” (Respondent 5, Male HCW).*

### Governmental support

For the government’s role in improving access and adherence to HU therapy, the healthcare workers suggested that the total cost of the therapy should be covered by the NHIS. This should include both the cost of the drug and the laboratory investigations required for monitoring the therapy. Additionally, adequate funding allocations should be earmarked to prevent stock-outs and maintain consistent medication supplies across healthcare facilities. Furthermore, the government should explore options to subsidize HU costs for patients, particularly those covered by health insurance schemes. This could involve negotiating preferential pricing agreements with pharmaceutical suppliers or incorporating HU into essential drug lists for subsidized procurement. Moreover, efforts should be made to enhance local manufacturing capacity for HU to reduce dependency on imported supplies and lower medication costs.

*“If you look at the challenges that were elucidated earlier, you notice that financial challenges rank high. So, I’m asking, you know, HU goes with some labs that are required to be done more frequently if a patient is on it. So, if HU is on insurance, then insurance should cover the lab. So those two should go hand in hand. If the government is absorbing the price of HU, this should go with the monitoring labs as well. The government and all experts involved should ensure that facilities have good stock of the medication at all times. And then of course, the government can also take up the responsibility of manufacturing HU within Ghana to ensure that we have a sustainable supply that is of high quality.” (Respondent 6, Female HCW).*

*“Well, we want stakeholders, especially the government, to make sure that from time to time we don’t run out of these medications. So, they have to deal directly with the insurance companies, so that they supply us with adequate stock to ensure that we don’t run out of stock” (Respondent 5, Male HCW).*

*Well, the first step has been taken, they’ve included it on any child’s benefit list and that’s fantastic. The next level would be to ensure that perhaps the companies that supply these medicines to the hospital get them at quite a competitive rate because many of these suppliers complain about the cost of NHIS fees, that what the insurance pays is too low. The government can look at it and increase the amount in order to meet the suppliers halfway so that they can keep supplying. Then obviously if it is possible for us to produce HU in the country, that will be superb so that the cost also reduces. Finally, they should be able to have the medication at different places across the country and not just in KATH so that patients can have access everywhere (Respondent 1, Male HCW).*

### Utilization and perception of mobile apps in enhancing healthcare services

Mobile apps have emerged as promising tools for enhancing healthcare services, including patient engagement, access to medical information, and disease management. The responses from HCPs shed light on the utilization and perception of mobile apps in the context of the Ahodwo CommCare mobile app in improving healthcare delivery and patient outcomes at the SCD clinic.

### Healthcare workers’ perspectives

Healthcare workers acknowledged the potential of mobile apps to revolutionize patient care and healthcare delivery. They recognized mobile apps as valuable tools for facilitating communication between HCPs and patients, enabling timely access to medical information and remote consultations. The respondents expressed that integration of mobile apps into healthcare practice can streamline administrative processes, such as appointment scheduling and medication reminders, enhancing operational efficiency and patient satisfaction. Moreover, HCWs interviewed emphasized the importance of patient education and empowerment through mobile apps. These platforms can serve as educational resources, providing patients with access to reliable health information, self-care resources, and lifestyle management tools. By empowering patients to take an active role in managing their health, mobile apps can contribute to improved treatment adherence and health outcomes.

### Healthcare workers’ utilization of Ahodwo CommCare mobile app

While some respondents reported using the app, a majority of the respondents expressed their lack of awareness, existence or functionality of the Ahodwo CommCare mobile app. When the respondents were asked to respond to the question “Do you use the Ahodwo CommCare mobile app at the SCD clinic? 1. Yes 2. No”. The following responses were recorded:

Yes (Respondent 1, Male HCW)

No, I don’t know about the app (Respondent 2, Female HCW)

No, I have not heard anything about it before (Respondent 3, Female HCW)

Yes, I do (Respondent 4, Female HCW)

No (Respondent 5, Male HCW)

Yes (Respondent 6, Female HCW)

*“Basically, the app was used for taking patient information and also for uploading and downloading their labs. And then if there were any toxic effects, we recorded and documented them on the app. It also served as a reminder for patients whose clinic days were due, to do their labs, etc. I mean, those were the things that we were using the mobile app for” (Respondent 6, Female HCW).*

**Fig 1:**
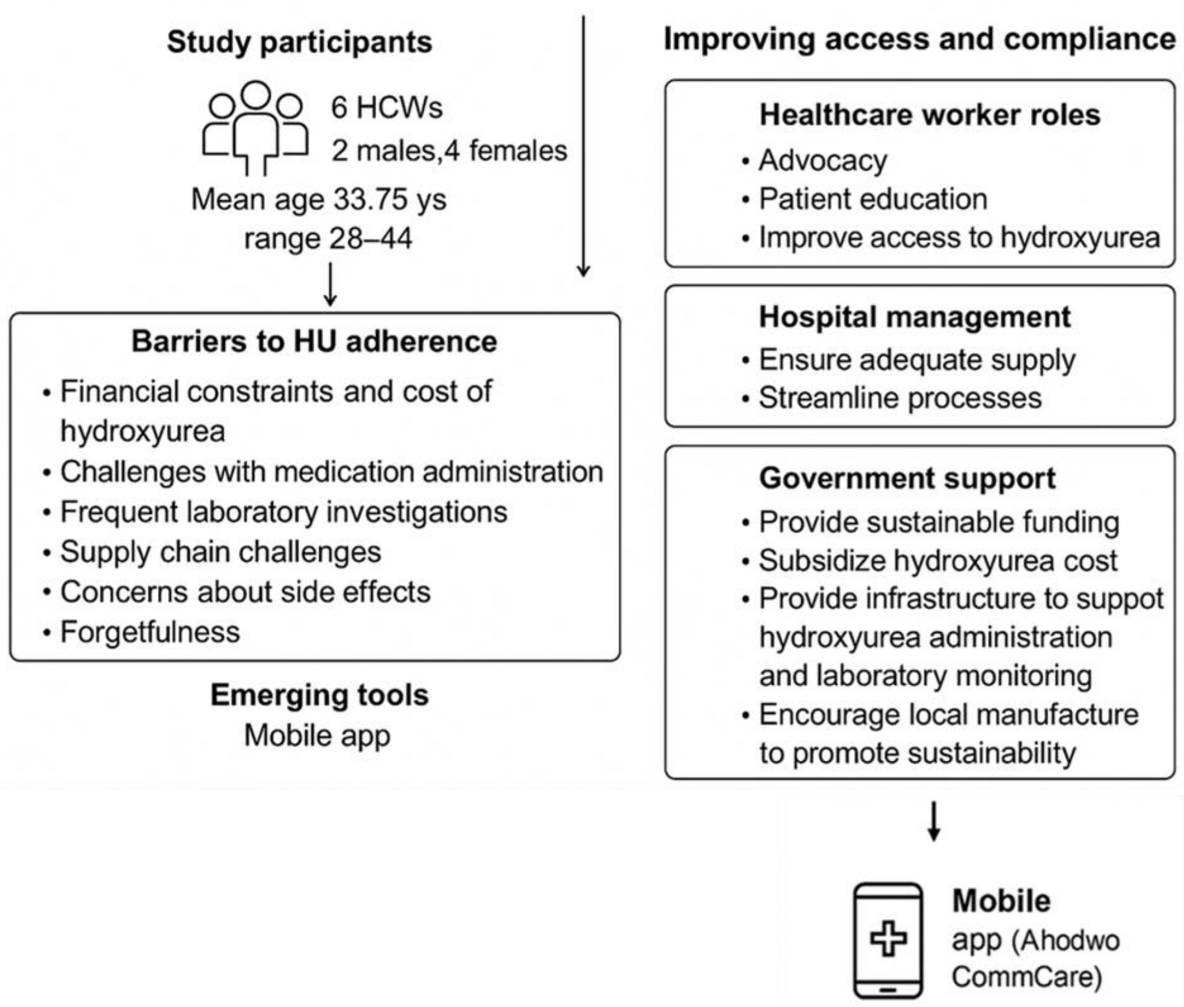
Summary of study findings: challenges and opportunities in hydroxyurea access and adherence for SCD Patients.

Those who used the app reported varying levels of utilization, with some indicating partial use for specific activities related to patient registration and data collection for the Ahodwo program. Among the respondents who did not use the app, the reasons cited for non-usage included lack of awareness about its existence and no prior knowledge of the app. Generally, mixed perceptions of the Ahodwo CommCare mobile app were observed, with a few respondents finding it moderately useful for data collection and patient reminders. This suggests a potential gap in communication or training regarding the implementation of the app within the clinic. This study further revealed a limited usage and awareness of other mobile apps at the KATH SCD clinic. Moreover, concerns regarding data privacy, security, and reliability may impact patient trust and acceptance of mobile health technologies (Fig 1).

*“So rarely, the mobile app was used when we were doing the Ahodwo program just to collect data for that program.” (Respondent 1, Male HCW).* *“The app was slightly useful” (Respondent 6, Female HCW).*

The diagram illustrates key themes identified from healthcare workers’ perspectives. Barriers to hydroxyurea adherence include financial constraints, challenges with medication administration, frequent laboratory investigations, supply chain challenges, concerns about side effects and forgetfulness. Improvement strategies are grouped under the roles of healthcare workers (patient education, advocacy and improvement of patient access to hydroxyurea), hospital management (ensuring adequate supply and streamlined distribution), and government support (sustainable funding, subsidizing cost of medication, infrastructural development and local production). Emerging tools such as the Ahodwo CommCare mobile app are highlighted as potential facilitators of adherence and improved patient monitoring.

## Discussion

Financial constraints emerged as a primary obstacle, aligning with previous research [29] that highlighted the financial burden associated with obtaining HU medication. Prior to its inclusion in the NHIS benefit list, patients had to pay out of pocket for the medication, posing a significant obstacle, especially considering the high cost of the medication. Even with its inclusion in the NHIS medicines list, issues with accessing medication persist, particularly when stock shortages occur especially towards the end of the year. Patients are then asked to seek for HU from pharmacies outside the hospital, adding complexity and inconvenience to the treatment process.

Similarly, the challenges related to stock shortages and medication availability resonate with various research findings [30], indicating that access barriers remain significant despite medication coverage under insurance schemes. In addition, fragmented national data on the SCD population in Ghana and treatment coverage have been identified as structural barriers, which make forecasting and procurement harder, thereby contributing to stock outs [29]. Therefore, strengthening supply chains and decentralizing HU services could help reduce stock outs [31].

Moreover, practical challenges in medication administration and concerns about side effects reported in our study compares well with other studies [30]. This highlights the importance of addressing patient education and allaying their fears regarding medication side effects. The HCPs indicated that patients tend to miss their medication doses due to forgetfulness or discontinue treatment when they experience adverse reactions or confusion regarding dosage instructions. Structured, pharmacist-led medication therapy management including counseling that normalizes side-effect monitoring and sets expectations can lessen fear-based nonadherence while aligning with dosing and monitoring of recommendations [31]. Improving access and compliance to HU among patients with SCD requires multifaceted interventions involving HCWs, hospital management, and government stakeholders. In our study, respondents highlighted the crucial role played by HCWs in educating patients and advocating for improved access to HU [32]. Regular patient education sessions provide opportunities to clarify misconceptions, reinforce the benefits of HU therapy, and address fears about side effects [33]. Evidence from implementation studies in Africa suggests that when patients understand the therapeutic benefits of HU including reduced frequency of vaso-occlusive crises and hospitalizations, their commitment to treatment improves substantially [30,34]. Furthermore, effective advocacy by HCWs can prompt administrative prioritization of HU procurement especially given that HU has been demonstrated to be a cost-effective intervention for reducing SCD complications [35].

Addressing practical and logistical barriers such as long waiting times for prescriptions and limited dispensing points within hospital settings as cited by respondents is essential to improving access. Streamlining the dispensing process through internal hospital distribution networks or specialized SCD pharmacy points can minimize patient delays and loss to follow-up. Some tertiary facilities in Nigeria and Kenya have successfully implemented dedicated SCD clinics or HU dispensing units that ensure predictable supply and timely refills [36]. Adopting a similar approach at KATH could enhance continuity of care and patient satisfaction.

Hospital management plays a pivotal role in facilitating access to HU. The call for streamlined administrative processes and timely medication procurement by our respondents echoes recommendations by Nguyen et al [37], underscoring the need for efficient healthcare delivery systems to enhance medication access. Hospital management must prioritize timely procurement of HU so that hospital pharmacies maintain uninterrupted stock. In sub-Saharan Africa, frequent supply disruptions of HU have been documented. The median availability score for HU was only 5/10, and 78% of respondents reported supply-chain disruptions for HU [38]. Such disruptions undermine patient trust and adherence. The hospital management must therefore design buffer‐stock policies, real‐time inventory monitoring, and streamlined requisition systems. To improve access and adherence to HU our respondents suggested that efforts should be made to negotiate favourable pricing agreements with suppliers to make HU more affordable for patients. This suggestion is very recommendable because in Ghana, HU has been included in the NHIS medicines list, which represents an important policy milestone for improving accessibility. However, in practice, the reimbursement prices quoted under NHIS are often lower than the prevailing market prices, creating a gap that discourages both pharmacies and distributors from stocking HU consistently. This discrepancy contributes to supply chain interruptions and places additional financial burden on patients who must pay out-of-pocket to cover price differences.

Governmental support, particularly in ensuring sustainable funding mechanisms and subsidizing medication costs, is equally consistent with the findings of Chen et al [39], which advocates for policy interventions to alleviate financial barriers to medication access. The alignment between this study’s findings and existing literature highlights the importance of collaborative efforts among stakeholders to address systemic barriers and improve medication adherence in SCD patients.

The Ahodwo CommCare mobile app was introduced at the SCD clinic at KATH to facilitate patient care and improve adherence to HU treatment. The findings indicate a mixed level of awareness and usage of the Ahodwo CommCare mobile app among HCPs at the SCD clinic. The study findings regarding the utilization and perception of mobile apps reflect a nuanced landscape, with similarities and differences compared to existing literature. While some HCPs reported using mobile apps for patient registration and data collection, others expressed limited awareness or concerns regarding data privacy, consistent with findings by Anderson et al. [40], highlighting the variability in HCPs attitudes towards mobile health technologies. Moreover, mixed perceptions on the mobile apps underscore the importance of addressing usability and trust issues, as emphasized by scholars who advocate for user-centered design and transparent data governance frameworks. However, the limited awareness and utilization of mobile apps among healthcare providers indicate the need for targeted training and integration efforts, aligning with recommendations by Roy et al. [41] to enhance HCP engagement with digital health tools.These findings present the importance of increasing awareness and promoting the usage of the Ahodwo CommCare mobile app among HCPs at the SCD clinic. To achieve this, training sessions should be conducted to educate providers about its features and benefits. Informational materials such as brochures and posters can equally raise awareness of the app’s capabilities. Hence, encouraging regular usage and integration of the app into daily clinical workflows is vital for maximizing its effectiveness in patient care and medication management.

### Strengths and Limitations

The semi-structured format of data collection was particularly advantageous as it allowed for flexibility, enabling interviewers to delve deeper into specific issues that participants raised. This approach not only facilitated the collection of rich qualitative data but also ensured that the interviews were dynamic and responsive to the unique insights of each participant.

Additionally, the in-depth interviews with HCW at the SCD clinic were conducted in a quiet and comfortable environment, recorded and transcribed for all feedback to be incorporated in study findings. Thematic analysis of the interviews allowed for an in-depth analysis of the HCWs viewpoints, enriching findings from the study. However, telephone interviews with some HCWs may have introduced some degree of context distortion, a possible limitation of the study.

## Conclusion

This study highlights the challenges that hinder effective HU access and adherence among patients with SCD at KATH. Financial constraints, medication stock-outs, administrative inefficiencies, and practical challenges in drug administration emerged as significant barriers. On the other hand, facilitators such as comprehensive patient education, proactive healthcare provider advocacy, improved internal distribution strategies, and the provision of governmental support were identified as critical components that could enhance adherence to HU therapy. The exploration of the Ahodwo CommCare mobile app further underscored its potential to streamline patient management by facilitating data collection, patient reminders, and appointment scheduling. However, limited awareness and inconsistent use among healthcare providers indicate that further training and integration efforts are necessary to harness the full benefits of digital health tools in this setting.

The findings highlight the need for coordinated, multi-level interventions that address both systemic and patient-specific challenges. Strengthening communication channels, streamlining administrative processes, securing sustainable funding, and enhancing digital literacy among healthcare providers can collectively improve HU adherence and overall SCD management.

## Supporting information

S1 File Transcript of Interview Responses. (DOCX)
