# Supplementary material for: Challenges and opportunities in hydroxyurea access and adherence for sickle cell disease patients at Komfo Anokye Teaching Hospital: Insights from healthcare providers
Source: PLoS One. 2026 May 29;21(5):e0347848. doi: 10.1371/journal.pone.0347848 (PMC13221035; doi:10.1371/journal.pone.0347848)
Supplement: S1 File — (DOCX) [file pone.0347848.s001.docx]

**Transcript 1**

**Speaker 1**: The next set is to look at hydroxyurea (HU) prescriptions and related factors. So, the first question is what medications are prescribed for sickle cell disease (SCD) patients at the SCD clinic?

**Speaker 2**: So, we have folic acid, penicillin V, zinc supplementation and HU, those are the routine medicines. Then any other intercurrent illnesses we can treat as and when a patients need them. It could be antibiotics or antimalarials.  But the routine ones are folic acid, penicillin V and usually zinc supplementation and HU.

**Speaker 1**: Ok, so with regards to the HU, what influences your choice for giving that medication to a patient.

**Speaker 2**: So, once the patient is at least nine months old and has sickle cell anaemia which is the SS genotype, we offer HU therapy for them.

**Speaker 1**: All right. So, in your opinion, what are some of the barriers to HU adherence among the patients?

**Speaker 2**: Oh, the biggest has always been about cost. Prior to it being included in the NHIS benefit list, it was a big problem because then they had to pay out of pocket for it. Then the next barrier will be access in terms of where they will find the medication because they wouldn't find it all the time within the hospital in Okomfo Anokye and they must go outside for it. The third one is for the children who cannot take capsules. The medication had to be reconstituted into suspension, and that came with added costs and added time because they had to also queue at the pharmacy to get that one done. Then finally, I mean for the minor ones, with either forgetfulness, they miss some doses because they forgot, or some felt that they were having some kind of side effects to the medicine and they stopped it or some were simply confused because they didn't get the right instructions, so they didn't come back for a refill when their first month, or their initial supply was done. Thank you.

**Speaker 1**: So, the next question is to look at how access can be improved, but we will look at the first part which is what healthcare workers can do to improve access then we will come to what hospital management can do and then look at the government. So, when we start with healthcare workers, in your opinion, what can be done by the healthcare worker at the SCD clinic to improve access and compliance to HU?

**Speaker 2**: I'm sure we can only advocate to both the patients and the community and tell them about the benefits of HU. And also advocate to the hospital management that the benefit goes beyond what the patients clinically get because when the patients benefit, they stay out of hospital and that means that there's lower cost of care for them and to the hospital. So we need to do more advocacy.

**Speaker 1**: Ok. And then the next one is to find out what the hospital can also do to improve access and compliance to HU.

**Speaker 2**: I guess if the management understands it, then they would have to stock it. I think that even if patients have to buy out of pocket, at least the hospital should be able to stock the medication. The hospital can stock generics of high quality so that the patient can trust the source, and that might also come at a lower cost compared to what is outside the hospital. So, the medicine must be available within the hospital, whether on insurance or off it. And that will only come when the management understands the benefits of the medication.

**Speaker 1**: Ok, so the last part of the question is to look at what can be done by the government to also improve access and compliance to HU.

**Speaker 2**: Well, the first step has been taken, they’ve included it on any child’s benefit list and that's fantastic. The next level would be to ensure that perhaps the companies that supply these medicines to the hospital get them at quite a competitive rate because many of these suppliers complain about the cost paid on NHIS, that the amount NHIS pays is too low, so maybe the government can look at this and increase the NHIS cost, and meet the suppliers halfway so that they can keep supplying. Then obviously if it is possible for us to produce it in the country then that will be superb so that the cost also comes down. And then finally, the medication should be available at different places across the country and not just in Okomfo Anokye so that patients can have access everywhere.

**Speaker 1**: Thank you. We want to look at the Ahodwo app. Do we use the ahodwo ComCare mobile app at the SCD clinic?

**Speaker 2**: Yes

**Speaker 1**: If yes, then if you use the Ahodwo ComCare mobile app at the SCD clinic, what is the level of use? Is it partial use or complete use?

**Speaker 2**: You know what, I will say partial, partial.

**Speaker 1**: Ok. What activities do you use the mobile app for?

**Speaker 2**: So rarely the mobile app was used when we were doing the Ahodwo program just to collect data for that program. So, the other side is that we still kept the hospital records in our electronic medical records system.

**Speaker 1**: Ok. Do you use it to interact with patients as in to contact patients?

**Speaker 2**: No, but I know nurses usually use it for appointments to remind them.

**Speaker 1**: Then in interacting with the patient, what activities do you use the mobile app to achieve?

**Speaker 2**: So personally, I've never used it for patients, but I do know that some patients reported that nurses with at least the app can send patients prompts that their clinic time was due. So that was nice.

**Speaker 1**: So, do you have an idea as to how many patients you use the app to reach out to in a week?

**Speaker 2**: With that one, I can't tell.

**Speaker 1**: Alright, to comment on the usefulness of the app in facilitating patient care at the SCD clinic, what would you say about this? It is a scale: not useful at all, slightly useful, moderately useful, very useful, and then extremely useful.

**Speaker 2**: I will say moderately useful.

**Speaker 1**: Have you ever used any other mobile app apart from the Ahodwo app?

**Speaker 2**: No

**Speaker 1**: Alright, thank you very much. That's all. Thank you.

**Speaker 2**: Alright. You are welcome

# **Transcript 2**

**Speaker 1**: What medications are often prescribed for SCD patients at the SCD clinic at Komfo Anokye?

**Speaker 2**: They are normally given folic acid, Pencillin V, Zincovit. and some are also given HU in addition to the three drugs I mentioned.

**Speaker 1**: OK. Have you ever recommended that HU is prescribed for a patient?

**Speaker 2**: No

**Speaker 1**: What is the reason for not recommending HU?

**Speaker 2**: I'm not a prescriber that is not my job description so I don't think it will good to be recommending HU for a patient. It all depends on the doctor.

**Speaker 1**: OK, alright. Do you think there are barriers to HU adherence among your patients.

**Speaker 2**: Yes

**Speaker 1**: OK. What are some of these barriers?

**Speaker 2**: Sometimes they complain that the drug is very very expensive. So If it's expensive and the patient is not well to do, I think it’s a bit difficult for them to purchase it.

**Speaker 1**: So, their major challenge is with their cost.

**Speaker 2**: Yes

**Speaker 1**: Alright. OK. Thank you. What can be done to improve access and compliance to HU? What can be done by the health worker? So, we will look at the health worker, hospital management, then we will look at the government. At these various levels, what can they do? We are starting with the healthcare worker, what can the health care worker do at the sickle cell clinics to improve access and compliance to HU?

**Speaker 2**: OK based on health worker, we can find a way to solicit for funds among ourselves, just to help the patients for a while, and then we'll talk to the management about it and see the way forward

**Speaker 1**: OK, I want to find out just what you said so. With, with soliciting for funds among the health workers do you think it is feasible. Do you think it is sustainable. What is your take on that?

**Speaker 2**: that is why I said for a while. Because I've encountered the situation where doctors and nurses and some medical staff contributed some of money to buy the medication for patients, just to support.

**Speaker 1**: In addition to that do you think there are other things that health workers can do?

**Speaker 2**: Well, I don't think so and I don't think it will be applicable because you can't support the patients for a very long time, you can do your best but this can't take you far.

**Speaker 1**: OK. With respect to what we are discussing, we are talking about the drug and the patient in totality, not only the affordability, but what whatever the patient needs to do in order to take that drug to be well, so from cost to adherence to everything. Do you think the healthcare worker has a role to play in that in that line?

**Speaker 2**: Yes, I think giving a health education or counselling the patient on the need for taking the drug without taking a break in it will help.

**Speaker 1**: Thank you. What do you think can be done by the hospital management to improve access and compliance to HU?

**Speaker 2**: well, I think. If there are ways and means the hospital is generating money, I think they should apportion some of the money to assist patients in getting these kinds of drugs. And then the education should also go, aside the education we should just maybe follow up or something just to help a patient take the drug and then investigate if they are taking it. If they are taking it, what is the effect and all that. I think that will help.

**Speaker** 1: I want to find out, do you know whether drugs are always available, whether the HU is always available at the pharmacy for the patients within the facility.

**Speaker 2**: If the medication is available, we get to know from the patients as most of the time the patients bring us feedback on whether they got the medication or not. Then we get to know that there is availability of the drug at the pharmacy.

**Speaker 1**: Most often, what report do you receive from the patient? Is the medication available or not available?

**Speaker 2**: It is available for a short period. Let's say if it's available, maybe for a month or two and sometimes, it can take a very long time.

**Speaker 1**: So, for this challenge, who can it be attributed to? Is it from the hospital management or from the government? Or where is it coming from based on your understanding.

**Speaker 2**: I think the drug is very expensive, so it being at the pharmacy in this facility, I think it was through the help of the government and the management of this hospital. They help in doing all that, but I think the drug is very costly and this is the problem. If the hospital secures HU, it is difficult for people to buy and it lasts for a very short time.

**Speaker 1**: Now we are moving on to the government. What do you think the government can do in order to improve access and compliance to HU?

**Speaker 2**: Government can enlist the medication on insurance. Even though the medication is not available, they can do that and then seek for help from elsewhere to get it. They have to consult doctors, pharmacists and know the way forward in getting this drug and making it available to the people.

**Speaker 1**: Thank you. I want to find out, do you use the Ahodwo Commcare mobile app at the SCD clinic?

**Speaker 2**: I don't use it

**Speaker 1**: Have you ever used it before?

**Speaker 2**: I don't know about the app

**Speaker 1**: You don't know about the app?

**Speaker 2**: I know of Ahodwo, the lab they were doing but I don’t know about the app

**Speaker 1**: Oh ok. You know about the labs they were doing, but you don't know about the mobile app.

**Speaker 2**: Yes

**Speaker 1**: OK. And do you know of any other app that maybe your facility or your clinic has ever used in terms of reaching out to patients, checking up on their medications, reminding them to come for clinic, etc.?

**Speaker 2**: I haven't heard of any app that does that.

**Speaker 1**: Alright. Thank you very much for your time, I'm grateful.

# **Transcript 3**

**Speaker 1:** The first question is what medications are often prescribed for sickle cell patients at the clinic. What kind of medications?

**Speaker 2:** Folic acid, Zincovit, HU

**Speaker 1:** Is that all?

**Speaker 2:** Yes,

**Speaker 1:** OK. So, the next one is, have you ever recommended that HU is prescribed for a patient?

**Speaker 2:** Yes,

**Speaker 1:** OK. What informed your recommendation? Why did you recommend it?

**Speaker 2:** It helps to prevent infections in the sickle cell patient and then I think it prevents crises, frequent crises too. I always recommend that they take their HU, when they come to the clinic.

**Speaker 1:** Thank you. So, in your opinion, what are the barriers to HU adherence in SCD patients?

**Speaker 2:** The patients have financial problems, the money in buying the drugs. I think the drugs are expensive.

**Speaker 1:** OK, so there are challenges, and some of the challenges are what?

**Speaker 2:** Financial

**Speaker 1:** OK.

**Speaker 2:** I think that's what the mothers/parents have been complaining about, that they don't have the money to buy the drugs.

**Speaker 1:** Alright. Is that all?

**Speaker 2:** So that's mostly the problem.

**Speaker 1:** The main factor is money to buy the drug?

**Speaker 2:** yes

**Speaker 1:** Alright we will be looking at three different questions. Firstly, we will look at the healthcare worker, Secondly, we will look at the hospital management, then the thirdly we will look at the government. We want to look at these 3 levels; the healthcare worker, at the facility level - the hospital management and then the government. What can they do to improve access and compliance to HU? We are starting with the first one. The first one is the healthcare worker. In your opinion, what can healthcare workers at the SCD clinic do to improve patient access and compliance to HU?

**Speaker 2:** I think you have to let the patient know the importance of taking the drugs. Well, if the patient knows the importance of taking the HU, I think he or she will always adhere to the drug. I think that's what the health worker can do. We can't do anything about it unless we tell the patients the importance of taking/giving them the drugs.

**Speaker 1:** The next one is what can be done by the hospital management to improve access and compliance to HU?

**Speaker 2:** I think the hospital can go and buy the drug, and then maybe reduce the price for the patient to buy from the pharmacy.

**Speaker 1:** Are you talking about the hospital pharmacy?

**Speaker 2:** Yes, the hospital pharmacy

**Speaker 1:** OK.

**Speaker 2:** I think if the hospital is going to buy the drug, they should buy in bulk. I think the hospital can buy the drugs in bulk and then reduce the prices for the patients so that when the patients come, they buy the medication from the hospital pharmacy. You know that the price will be low as a result.

**Speaker 1:** OK, so if I get you well, are you are suggesting that the hospital should buy the drugs so that the patient can get it at a reduced price? Does that mean that some of the patients don't access the drugs here? Do they go out?

**Speaker 2:** Yes, most of them

**Speaker 1:** Most of them go out because it's not available at the facility?

**Speaker 2:** Yes

**Speaker 1:** Alright. The next one is what can be done by the government to improve access and compliance to HU.

**Speaker 2:** I think they can add the drug to the health insurance list, right.

**Speaker 1:** The HU is now on the health insurance.

**Speaker 2:** But the patients don’t get some of the drug when they get to the pharmacies.

**Speaker 1:** It is on health insurance now.

**Speaker 2:** They said it is on health insurance but when the patient comes and you tell them to go for the drug at the hospital pharmacy, they will tell the patients the drugs are not available. That they should go outside, to a pharmacy outside they will get it there. The patients are stressed already so they will go home. They will not even go and buy the drug.

**Speaker 1:** OK, so what do you suggest that government should do to improve access and compliance to HU?

**Speaker 2:** If HU will be free like the HIV and TB medications, I think it will help.

**Speaker 1:** OK, alright. We are moving on to the next one. Do you use, or have you used the Ahodwo Comcare mobile app at the SCD clinic before?

**Speaker 2:** I have not heard anything about it.

**Speaker 1:** OK, so you haven't used any app to care for patients to schedule their visits. You haven't used anything like that before. OK.

**Speaker 2:** The response is negative.

**Speaker 1:** If you haven't used it before, why? Is it because the app being used at the clinic and you don't have access to using it or it is not there at all. I want to know the reason why you haven't used it before or you don't use it.

**Speaker 2:** Maybe they have. But I don't have any idea about.

**Speaker 1:** OK. OK, so you don't know whether there is an app there or not.

**Speaker 2:** Yes

**Speaker 1:** Ok alright. Have you used any different app in scheduling for patients or in doing something for patients before? Not Ahodwo, but any other mobile app to interact with patients? Have you used anything like that before?

**Speaker 2:** At work?

**Speaker 1:** Yes, at the clinic. A mobile app on the phone which you have used to interact with patients before.

**Speaker 2:** mmmm, yes

**Speaker 1:** Which app is that? Can you can remember the name?

**Speaker 2:** (smiles, while trying to remember)

**Speaker 1:** Do you still use that app?

**Speaker 2:** No

**Speaker 1:** Ok. When did that happen? When did you use the app? Was it one year ago, two years ago, three years ago?

**Speaker 2:** Two, years.

**Speaker 1:** Two years ago, what did you use the app for? I meant an app that has been developed specifically for sickle cell patients.

**Speaker 2:** No, no, no. no, no, no, no.

**Speaker 1:** So, the answer is no you haven't used any other app before. Ok.

**Speaker 1:** Then I think that is all because you haven't used it before so the other questions are not applicable to you. Thank you very much for your time. I'm grateful.

# **Transcript 4**

**Speaker 1**: What medications are often prescribed for Sickle cell patients.

**Speaker 2**: I think the most common ones are folic acid, Penicillin V, and Zincovit. That's the most common one.

**Speaker 1**: Have you ever recommended HU to a patient?

**Speaker 2**: Almost all the time to the SS genotypes.

**Speaker 1**: What influenced your decision to recommend HU to the patients?

**Speaker 2**: I've realized that almost all the kids who are on it hardly get crises. It's been a while. I can confidently say even this year I’ve not even had one student or a child who is on HU that has gone into crisis. So, I am all the time recommending it for the parents if they are already not on it.

**Speaker 1**: What are the barriers to HU adherence in patients.

**Speaker 2**: Eerh money, Because of the costs, most of the parents find it difficult buying especially those who have more than one child with SS genotype, they have difficulties buying their drugs. And aside that, sometimes when they are not able to come for appointment they stop taking the medicines, especially when the medicine gets finished along the way. They stop taking the medicine until they come back for the doctor to prescribe. And at times if they go to get refills on insurance and the medication is not available, they find it difficult buying. So, they stop taking the medicine until they are able to raise money to buy. So, money is the main barrier to the administration of the drug.

**Speaker 1**: Alright, thank you. The next question - we want to find out in your opinion what can be done by the healthcare workers at the SCD clinic to improve access and compliance to HU.

**Speaker 2**: Access I wouldn't say there is much we can do. We can only educate with the compliance. Tell them the implication of not following the regimen. Tell them how they will benefit when they take the medication, when they follow the regimen keenly. But then for access, if they come and they are not able to buy the medicine, sometimes it's difficult. You can't buy for all the patients, even if you would want to be kind to them. Sometimes they can only afford a month and can't buy for more than a month, and if it gets finished, it's finished. If they all don't have money to go and buy, there is nothing you can do. Maybe the government can come in.

**Speaker 1**: The next question is what can the hospital management do to improve access and compliance to HU? What do you think can be done by the hospital management improve access to compliance to HU.

**Speaker 2**: For the hospital management, if the medicine is at the hospital, they should cut down some of these bureaucracies and make it available at the Pharmacies so that when the patients go to the pharmacies, they will have access to the medication.

**Speaker 1**: Ok. So, it means that there are bureaucracies when it comes to accessing the medication by the patients.

**Speaker 2**: Yes, yes, a whole lot of it. Someone signing, someone checking…. So sometimes it may be in but it will take like a week before they are distributed to the pharmacies, which if a patient comes within that week, it means they won’t have access to it. And once the patient leaves the hospital, they are gone until the next review. They can't have access to the medication

**Speaker 1**: Thank you, the next part is to look at what can be done by the government to improve access and compliance to HU.

**Speaker 2**: I don't know how the government is funding it, but I believe the funds are not coming in as is supposed to be. This is because sometimes when the medication comes in, less than three weeks it gets finished. That means whatever amount they bought is not enough because in a week we see more than almost 200 patients. And if just three weeks, everything gets finished, it means that the medication is not enough.

**Speaker 1**: We will look at the Ahodwo Comcare mobile app

**Speaker 2**: I have not used it before.

**Speaker 1**: You have not used it before…oK.

**Speaker 2**: I have the Ahodwo phone but I don't know If that's what it is. I have that Ahodwo phone that we use for the registration of the patients on HU. I don’t know if that is the app

**Speaker 1**: I'm sure yes, that is it.

**Speaker 1**: Alright, so you use the Ahodwo Comcare mobile app.

00:06:37 **Speaker 2**: Yes, I do, yes.

**Speaker 1**: You do alright, so what is your level of usage?

**Speaker 2**: Partial

**Speaker 1**: Partial, what activity do you use the mobile app?

**Speaker 2**: Registering patients on HU and sometimes finding information about them.

**Speaker 1**: You use it for registering the patient for….

**Speaker 2**: Those who are on HU, like the newly recruited ones. And then if you want information about any of the old patients, you can get it from the app.

**Speaker 1**: Ok. Do you use the app to interact or contact the patients?

**Speaker 2**: No, I have not done that before.

**Speaker 1**: You haven’t done that before?

**Speaker 2**: I would go to the app and get their information, sometimes their numbers to call them, but I don't contact them directly through the app.

**Speaker 1**: Alright. Comment on the usefulness of the app in facilitating patient care at the clinic. There are responses to select for this; not useful at all, slightly useful, moderately useful. very useful and then extremely useful

**Speaker 2**: Moderately

**Speaker 1**: Moderately useful... ok. So have you ever used any mobile app other than the Ahodwo Comcare app?

**Speaker 2**: In terms of medical, yes, yes.

**Speaker 1**: Ok, so which app is that?

**Speaker 2**: Uh, what is the name? med pr … or something something. I have actually forgotten the name? But I have used it.

**Speaker 1**: You have used one before, ok.

**Speaker 2**: It is for medications. The one I'm talking about is for medications and it's not specifically for sickle cell, but general health.

**Speaker 1**: This is the end of our interview, thank you very much for your time.

**Speaker 2**: You're welcome. You're welcome.

**Speaker 1**: I’m grateful.

# **Transcript 5**

**Speaker 1**: What medications are often prescribed for Sickle cell patients at the sickle Cell unit of Okomfo Anokye Teaching Hospital?

**Speaker 2**: The medications which are usually prescribed for sickle cell patients at the Okomfo Anokye Teaching Hospital are HU or hydroxycarbamide, folic acid, Zincovit and then penicillin V

**Speaker 1**: Ok. Alright. The next question is, have you ever recommended that HU is prescribed for a patient?

**Speaker 2**: Yes

**Speaker 1**: OK. What influenced your decision to recommend HU to be prescribed for them? What influenced your decision?

**Speaker 2**: For the time of being here, I've realized that for most patients especially those who are with the SS genotype, some of them usually present with more frequent crises and most of them were on admission, Because of recurrent vaso-occlusive pain crises or episodes. But then, since the introduction of HU, you realize that there has been a decrease in hospitalizations due to possible reduction in vaso-occlusive pain episodes. So that is the reason why I recommended HU for patients who usually come with more frequent crises.

**Speaker 1**: OK. In terms of prescribing HU for the SCD patients and its usage, have you witnessed or seen any barriers that affect the patient adherence to the medication?

**Speaker 2**: Yes, yes, yes, yes. The main barrier has to do with finances, because previously it wasn't insured, so patients would have to buy out of pocket. And you know, in our part of the world especially in sub-Saharan Africa and with Ghana being part of the low to middle income countries, most patients usually find it difficult to raise money for this expensive drug. But then for now it is insured, so most patients access it for free.

**Speaker 1**: Ok, so with the with the insurance, now it is on insurance. Now that it is on insurance, do the patients have access to it?

**Speaker 2**: Yes, they have access to it.

**Speaker1**: They don’t face any kind of challenges?

**Speaker 2**: Oh, no, no, no. The only time they may have a challenge is when we are out of stock. Even for that one, we issue prescription cards for them so that they can access it for free in pharmacies which are National Health Insurance Scheme (NHIS) accredited.

**Speaker 1**: Alright, so the next question we want to find out what can be done by healthcare workers at the SCD Clinic to improve access and compliance to HU. So that's what we are looking at; the healthcare workers. What can you do at the clinic to improve access and compliance to HU?

**Speaker 2**: We have to make sure that we always have enough stock of the medications so that at least every patient which goes to the pharmacy units will have access to these medications because whenever there is denial of these medications, defaults may start to come in, and of course negatively it will have an impact on the patient outcomes. And adherence can also be captured in this manner.

**Speaker 1**: Now we also want to look at what can be done by the hospital management to improve access and compliance to HU.

**Speaker 2**: For now, the only place you can access HU is the specialist OPD pharmacy. So, I think to improve access the scope should be widened. Other units should also be given the opportunity to serve these medications so that they don't queue for a very long time before they can access HU for free.

**Speaker 1**: Alright. And then next, we want to find out what can be done by the government to improve access and compliance to HU.

**Speaker 2**: Well, we want stakeholders, especially government, to make sure that from time to time we don't run out of these medications. So, they have to deal directly with the insurance companies, so that they supply us with adequate stock so that we don't run out of stock.

**Speaker 1**: So has it ever happened that, you know, for a long time or for a period of time there was no HU at Okomfo Anokye.

**Speaker 2**: Oh yes yes, it happens sometimes. Getting towards the end of the year we ran out of stock of HU where patients had to access it outside.

**Speaker 1**: OK. Do you have access to Ahodwo Comcare mobile app at your place?

**Speaker 2**: No.

**Speaker 1**: What is or are the reasons for not for not using it.?

**Speaker 2**: I'm not aware of this app anyway.

**Speaker 1**: Alright, so it means you haven't used the app before.

**Speaker 2**: Yes, yes, yes, yes.

**Speaker 1**: I want to find out do you think usage of an app in terms of communication between patients and their doctors, nurses and pharmacists can be a way of improving access? Do you think something of that could be of importance?

**Speaker 2**: Yes, yes it will, yes, yes.

**Speaker 1**: Thank you very much for your time and I'm very grateful. Thank you.

**Speaker 2**: You're welcome.

**Transcript 6**

**Speaker 1:** Okay, thank you. Now we are moving on to HU prescription and app-related factors. What medications are often prescribed for SCD patients at the SCD clinic?

**Speaker 2:** So, we give the routine medications, which are folic acid, penicillin V, Zincovit, and if indicated, HU.

**Speaker 1:** Okay, alright. So with regards to HU, what factors influence the choice for that? Why do you prescribe that for patients?

**Speaker 2:** It depends on the kind of sickle cell the patient has, that is, the phenotype of the patient. It also depends on the age of the patient. If the patient is within the age bracket, and it also depends on the caregiver. If they are willing to put in the commitment that HU administration requires, then of course the patient can be offered HU.

**Speaker 1:** Alright, thank you. The next question is: In your opinion, what are the barriers to HU adherence in patients?

**Speaker 2:** For adherence, the main barrier, looking at where we are located, a low-middle-income country, is financial challenges. That is the main barrier. The second barrier is patient-dependent. Some people have misconceptions when it comes to the usage of HU, and based on those misconceptions, they may not adhere to taking it. Another barrier is the frequent laboratory investigations required during HU therapy. Some parents are uncomfortable with repeated needle pricks in their children, and based on that, they are reluctant to come frequently. Another barrier is inconsistent supply. Even though HU is supposed to be on the NHIS, we do not usually have continuous supply. Patients who cannot afford it are unable to get it when it is not in stock. Side effects are also a barrier. If a patient experiences side effects more than once, they may be reluctant to continue. Another barrier is misinformation from some health professionals due to inadequate knowledge, which can discourage adherence.

**Speaker 1:** Alright, thank you very much. The next question is: What can healthcare workers do to improve access and compliance to HU?

**Speaker 2:** Healthcare workers should provide proper education, information, and communication. They should address misconceptions and create a friendly clinic environment to encourage openness. They should also train themselves to be qualified HU prescribers so that they can improve access under the NHIS.

**Speaker 1:** What can hospital management do to improve access and compliance to HU??

**Speaker 2:** Hospital management should ensure consistent supply and source HU from well-established pharmaceutical companies. Bulk purchasing and partnerships can help reduce cost. They should also ensure stock availability all year-round and collaborate with other facilities to improve access. Additionally, local production by hospital pharmacists can be explored.

**Speaker 1:** What can the government do to improve access and compliance to HU?

**Speaker 2:** The government should ensure that both HU and its required laboratory tests are covered under NHIS. They should also ensure consistent supply and support local manufacturing to improve sustainability and quality.

**Speaker 1:** Do you use the Ahodwo Comcare mobile app?

**Speaker 2:** No, not currently. Previously we used it, but now we do not due to lack of supply support and absence of a data manager.

**Speaker 1:** What activities was the app used for?

**Speaker 2:** It was used for patient information, lab recording, toxicity monitoring, reminders for clinic visits, and communication with patients.

**Speaker 1:** How useful was the app?

**Speaker 2:** Slightly useful.

**Speaker 1:** Have you used any other mobile app?

**Speaker 2:** Yes.

**Speaker 1:** Thank you very much. This is the end of the interview.
